# Supplementary material for: MetaRibo-Seq measures translation in microbiomes
Source: Nat Commun. 2020 Jun 29;11:3268. doi: 10.1038/s41467-020-17081-z (PMC7324362; doi:10.1038/s41467-020-17081-z)
Supplement: Supplementary file 10 — Supplementary Data 7 [file 41467_2020_17081_MOESM10_ESM.zip › File2/Confidence_High_Taxonomy/144820_out.krona.html]

Javascript must be enabled to view this page.

members
magnitude
magnitudeUnassigned
count
unassigned
taxon
rank

144820\_out

2

2
2
superkingdom

1
1239
phylum

186801
1
class

order
186802
1

family
186803
1

genus
1
265975

1
1501329
species

SRS149671\_contig\_number\_contig-100\_1468.226024

1
32066
phylum

203490
1
class

1
203491
order

1
1129771
family

genus
1
32067

712357
1

SRS065099\_contig\_number\_contig-100\_925.172608
species
